# Clinicopathological characteristics and optimal management for esophagogastric junctional cancer; a single center retrospective cohort study

**DOI:** 10.1186/1756-9966-32-2

**Published:** 2013-01-07

**Authors:** Hiroaki Ito, Haruhiro Inoue, Noriko Odaka, Hitoshi Satodate, Michitaka Suzuki, Shumpei Mukai, Yusuke Takehara, Hiroyuki Kida, Shin-ei Kudo

**Affiliations:** 1 Digestive Disease Center, Showa University Northern Yokohama Hospital, 35-1 Chigasakichuo Tsuzuki-ku, Yokohama, 224-8503, Japan

**Keywords:** Esophagogastric junctional cancer, Esophageal cancer, Gastric cancer, Lymph node metastasis

## Abstract

**Background:**

Esophagogastric junctional (EGJ) cancer occurs in the mucosa near the esophagogastric junction, and has characteristics of both esophageal and gastric malignancies; its optimal treatment strategy is controversial.

**Methods:**

We conducted a single-center retrospective cohort study of the patients who underwent curative surgery with lymphadenectomy for EGJ cancer. Tumor specimens were categorized by histology and location into four types—centered in the esophagus < 5 cm from EGJ (type E), which were subtyped as (i) squamous-cell carcinoma (SQ) or (ii) adenocarcinoma (AD); (iii) any histological tumor centered in the stomach < 5 cm from EGJ, with EGJ invasion (type Ge); (iv) any histological tumor centered in the stomach < 5 cm from EGJ, without EGJ invasion (type G)—and classified by TNM system; these were compared to patients’ clinicopathological characteristics and survival outcomes.

**Results:**

A total of 92 EGJ cancer patients were studied. Median follow-up of surviving patients was 35.5 months. Tumors were categorized as 12 type E (SQ), 6 type E (AD), 27 type Ge and 47 type G; of these 7 (58.3%), 3 (50%), 19 (70.4%) and 14 (29.8%) and 23 patients, respectively, had lymph node metastases. No patients with type E (AD) and Ge tumors had cervical lymph node metastasis; those with type G tumors had no nodal metastasis at cervical and mediastinal lymph nodes. Multivariate analysis showed that type E (AD) tumor was an independent prognostic factor.

**Conclusions:**

We should distinguish type Ge tumor from type E (AD) tumor because of the clinicopathological and prognostic differentiation. Extended gastrectomy with or without lower esophagectomy according to tumor location and lower mediastinal and abdominal lymphadenectomy are recommended for EGJ cancer.

**Trial registration:**

University Hospital Medical Information Network in Japan, UMIN000008596.

## Background

Gastric and esophageal cancers are, respectively, the fourth and eighth most common cancers in the world, and the second and sixth most common causes of cancer-related death, affecting approximately 736,000 and 406,000 people in 2008 [1]. Esophagogastric junctional cancer (EGJC), which is increasing in Western countries, is a tumor occurring at the mucosa between the lower esophagus and cardia, and has clinicopathological characteristics of both esophageal and gastric malignancies [2,3].

Siewert classification is widely used to categorize EGJ adenocarcinoma [4,5]. Siewert defines adenocarcinoma of the distal esophagus, such as that from specialized esophageal metaplasia (e.g., Barrett’s esophagus) as type I; cardiac carcinoma, from the cardia epithelium or within 1 cm (along the esophagus) or 2 cm (in the stomach) from the EGJ as type II; and subcardial gastric carcinoma with epicenter in the proximal 5 cm of the stomach, which infiltrates the EGJ and distal esophagus, as type III. Because the Siewert type I tumor is located in the lower esophagus, it can be treated as lower esophageal cancer; whereas type III tumor has similar clinicopathological characteristics to cardiac cancer because of its location. However, Siewert type II tumor is a metastatic threat to both thoracic and abdominal areas, as it crosses the EGJ. Subtotal esophagectomy offers only a limited benefit and should not be performed for type II cancer. The TNM staging system according to the seventh edition of the American Joint Committee on Cancer/International Union Against Cancer (AJCC/UICC) Cancer Staging Manual defined EGJC, including of squamous-cell carcinoma and adenocarcinoma centered in the esophagus within 5 cm, and in the proximal 5 cm of the stomach with crossing the EGJ [6,7]. AJCC/UICC also categorizes any cardiac cancer without EGJ invasion as gastric cancer regardless of its location. Different staging systems are applied to esophageal squamous-cell carcinoma and esophageal adenocarcinoma.

Surgery is effective treatment for resectable esophageal [8,9] and gastric cancer [10-12]. However, as esophagectomy is generally more invasive than gastrectomy [13], we should be careful in treating EGJC with esophagectomy. We studied clinicopathological characteristics of patients with EGJC to investigate its optimal management.

## Methods

### Study design

We performed a single center, retrospective cohort study. We studied patients who underwent curative surgery for EGJC, including lymph node dissection, at the Digestive Disease Center, Showa University Northern Yokohama Hospital, between October 2001 and December 2010. Clinicopathological data and prognosis were taken from medical records.

### Patients

We studied patients with cancer in the lower esophagus and cardia. Inclusion criteria were: (i) presence of histologically proven carcinoma centered within the lower 5 cm of the esophagus and the upper 5 cm of the stomach; (ii) clinically solitary tumors; (iii) no prior endoscopic resection or surgical treatment; and (iv) patient aged 20–80 years. The exclusion criteria were: (i) presence of severe organ dysfunction; (ii) presence of metachronous and synchronous malignancy; and (iii) presence of pathological non-curative findings.

All patient data were approved for use by the institutional review board of Showa University Northern Yokohama Hospital. This study was registered with the University Hospital Medical Information Network in Japan (No. UMIN000008596).

### Classification

**Figure 1:**
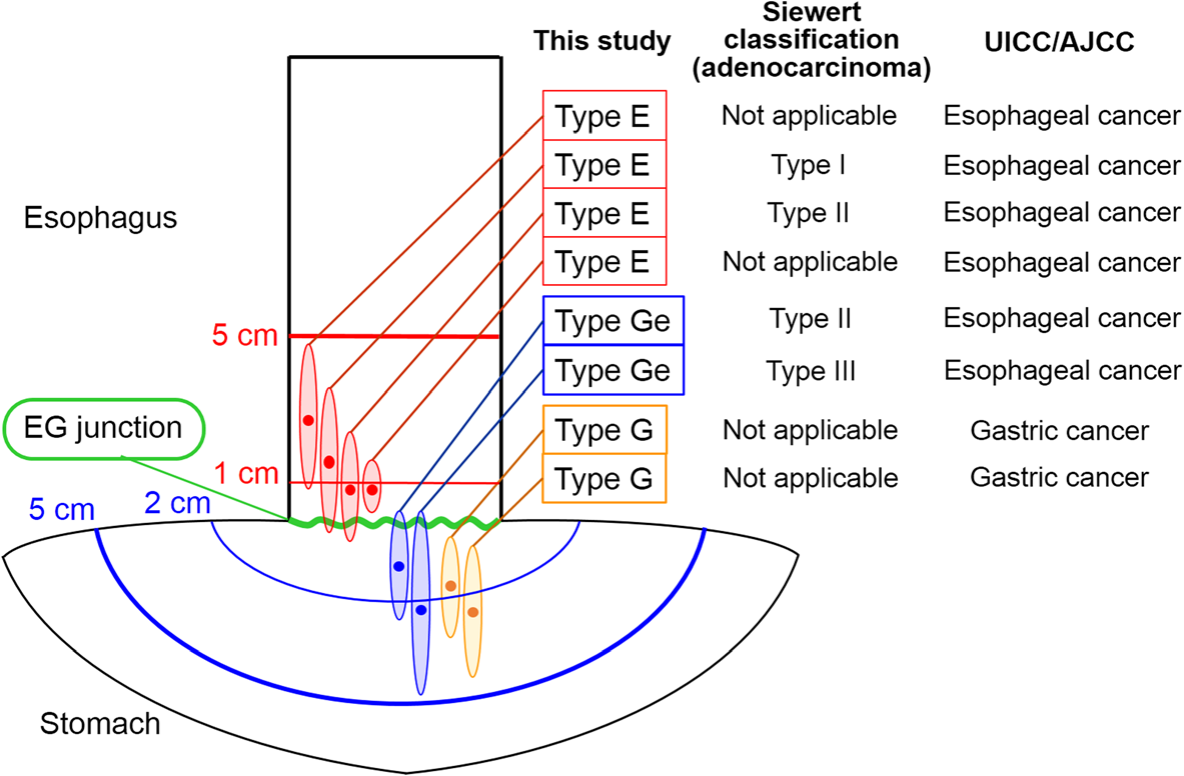
**Tumor classification.** We categorized tumors near the EGJ into four types according to its location and main histological type. Categorization criteria were: (**i**) squamous-cell carcinoma with epicenter in the esophagus within 5 cm from EGJ (type E (SQ)); (**ii**) adenocarcinoma with epicenter in the esophagus within 5 cm from EGJ (type E (AD)); (**iii**) any histological tumor with epicenter in the stomach within 5 cm from EGJ, with EGJ invasion (type Ge); (**iv**) any histological tumor with epicenter in the stomach within 5 cm from EGJ, without EGJ invasion (type G). Type E (SQ), E (AD) and Ge tumors were categorized as esophageal cancer; type G tumor was categorized as gastric cancer by the American Joint Committee on Cancer/International Union Against Cancer (AJCC/UICC) Cancer Staging Manual. Siewert type I and III tumors were categorized as type E (AD) and Ge tumors, and Siewert type II tumor was categorized as type E (AD) or Ge tumor in this study.

Although Siewert classification is one of the most widely used criteria for EGJC, it is generally used for only adenocarcinoma. EGJC, including squamous cell carcinoma, has been defined by the seventh edition of AJCC/UICC TNM Cancer Staging Manual. However, it does not cover all of the cancer near the EGJ—for example a localized gastric adenocarcinoma with centered in the stomach within 5 cm from EGJ. Thus, we categorized tumors near the EGJ into four types, according to location and main histological type (Figure 1). Categorization criteria were: (i) squamous-cell carcinoma centered in the esophagus within 5 cm from EGJ (type E (SQ)); (ii) adenocarcinoma centered in the esophagus within 5 cm from EGJ (type E (AD)); (iii) any histological tumor centered in the stomach within 5 cm from EGJ, with EGJ invasion (type Ge); (iv) any histological tumor centered in the stomach within 5 cm from EGJ, without EGJ invasion (type G). All disease was pathologically staged using the seventh edition of AJCC/UICC TNM Cancer Staging Manual [6,7]. Thus, types E and Ge tumors were staged as esophageal cancer, and type G tumor was staged as gastric cancer.

### Statistical analysis

Statistical analysis was performed using JMP 9.0.3 (SAS Institute, Cary, USA). We used Fisher’s exact test and Pearson’s chi-squared test to compare the characteristics of the patients and pathological findings. The nonparametric Kruskal–Wallis test was used to assess differences among patients’ age groups, number of dissected lymph nodes and pathological tumor size. Kaplan–Meier curves of estimated overall survival were generated and compared, using a 2-sided log-rank test. To investigate prognostic factors, Cox proportional hazard analysis was used. Multivariate analysis included tumor types and variables with *P* < 0.10 in univariate analysis. *P* < 0.05 was considered statistically significant.

## Results

### Patient characteristics

**Figure 2:**
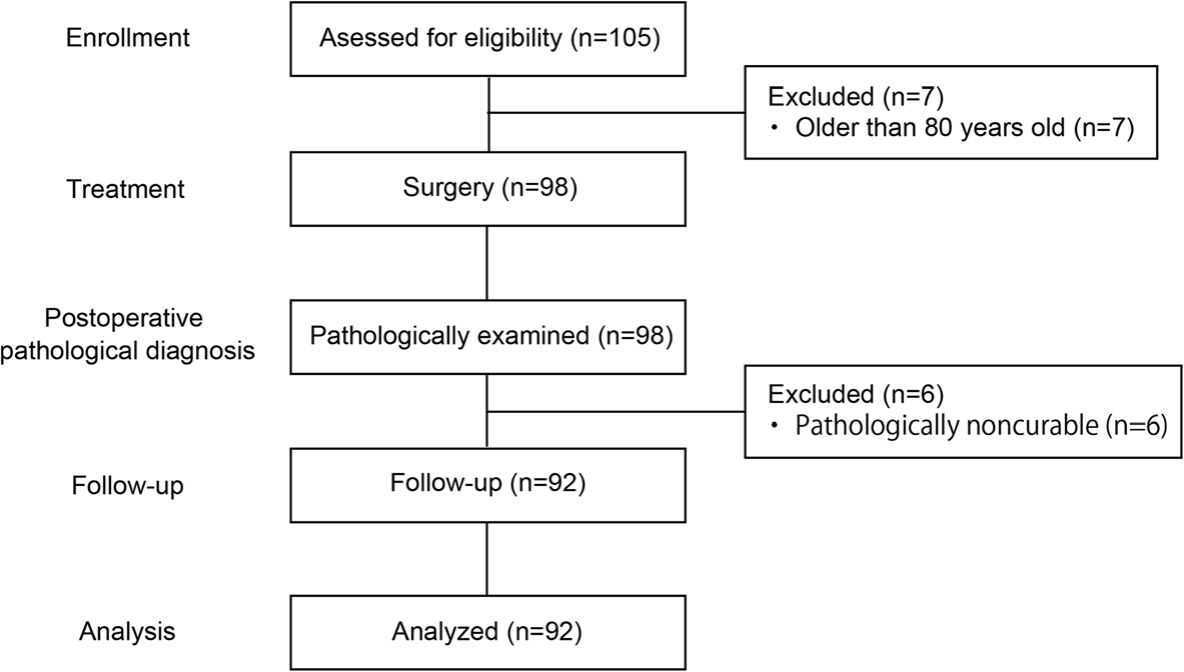
**Flow diagram of the patients in this study.** Total 92 patients who underwent curative surgical resection for esophagogastric junctional cancer at the Digestive Disease Center, Showa University Northern Yokohama Hospital between October 2001 and December 2010 were retrospectively studied.

A total of 92 patients were included in this study (Figure 2). Median follow-up of surviving patients was 35.5 months. Patients’ characteristics are summarized in Table 1. Approximately 80% of them were men; their average age was 65.9 years (range: 35–80 years). Fourteen (15.2%), 30 (32.6%) and 48 (52.2%) patients underwent subtotal esophagectomy with partial gastrectomy, proximal gastrectomy with partial esophagectomy and total gastrectomy with partial esophagectomy, respectively. Twenty-four patients underwent splenectomy to remove involved lymph nodes at the splenic hilum. Thirteen patients (14.1%) received preoperative chemotherapy. Histologically, 79 (85.9%) and 13 (14.1%) of 92 patients had tumors mainly composed with adenocarcinoma and squamous cell carcinoma. Mean pathological tumor size was 46.1 mm. Two, 16 and 11 tumors were categorized as Siewert types I, II and III, respectively; Siewert classification was not applicable to the remaining 63 tumors. In 63 tumors which did not apply to Siewert classification, 50 and 13 tumors were mainly composed with adenocarcinoma and squamous cell carcinoma. However 15 and 48 tumors centered in the esophagus and the stomach, only one tumor had esophagogastric junctional invasion. Eighteen (19.6%), 27 (29.3%) and 47 (51.1%) tumors were categorized type E, G and Ge, respectively. The mean number of dissected lymph nodes was 37.2 ± 28.0 (SD) in each patient. Forty-five (48.9%) of 92 patients had lymph node metastases (pN1–3). Thirty-six (39.1%), 19 (20.7%), 17 (18.5%) and 20 (21.7%) patients were pathologically staged I, II, III and IV, respectively. Forty-nine patients (53.3%) had preoperative chemotherapy.

**Table 1:** Patient characteristics (n = 92)

	**Variables**	
Age (year, mean ± SD)		65.9 ± 9.4
Sex	Male	72 (78.3%)
	Female	20 (21.7%)
Siewert classification	Type I adenocarcinoma	2 (2.2%)
	Type II adenocarcinoma	16 (17.4%)
	Type III adenocarcinoma	11 (12.0%)
	Not applicable	63 (68.5%)
Macro type	Type 0	36 (39.1%)
	Type 1	4 (4.3%)
	Type 2	26 (28.3%)
	Type 3	21 (22.8%)
	Type 4	1 (1.1%)
	Type 5	4 (4.3%)
Preoperative chemotherapy	No	79 (85.9%)
	Yes	13 (14.1%)
Extent of surgical resection	Subtotal esophagectomy with partial gastrectomy	14 (15.2%)
	Proximal gastrectomy with partial esophagectomy	30 (32.6%)
	Total gastrectomy with partial esophagectomy	48 (52.2%)
Extent of lymph node dissection	Abdominal, mediastinal and cervical	11 (12.0%)
	Abdominal and mediastinal	9 (9.8%)
	Abdominal and lower mediastinal^†^	27 (29.3%)
	Abdominal	45 (48.9%)
Pathological tumor size (mm, mean ± SD)		46.1 ± 23.7
Main histologic type	Adenocarcinoma	79 (85.9%)
	Squamous-cell carcinoma	13 (14.1%)
Lymphatic invasion	L0	32 (34.8%)
	L1	60 (65.2%)
Venous invasion	V0	32 (34.8%)
	V1–2	60 (65.2%)
Pathological depth of tumor invasion	pT1	33 (35.9%)
	pT2	11 (12.0%)
	pT3	35 (38.0%)
	pT4	13 (14.1%)
Lymph node metastasis	pN0	47 (51.1%)
	pN1	19 (20.7%)
	pN2	14 (15.2%)
	pN3	12 (13.0%)
Distant metastasis	pM0	72 (78.3%)
	pM1	20 (21.7%)
TNM stage	pStage I	36 (39.1%)
	pStage II	19 (20.7%)
	pStage III	17 (18.5%)
	pStage IV	20 (21.7%)
Adjuvant chemotherapy	No	43 (46.7%)
	Yes	49 (53.3%)

^†^ Including lower thoracic paraesophageal, diaphragmatic and posterior mediastinal lymph node.

Comparison of clinicopathological characteristics among type E (SQ), E (AD), Ge and G tumor group are summarized in Table 2. There were significant differences in extent of surgical resection, pathological tumor size, main histological type, depth of tumor invasion (pT category), lymph node metastasis (pN category), distant metastasis (pM category) and TNM tumor stage. Histologically, 26 (96.3%) of 27 type Ge tumor and all 47 type G tumors were adenocarcinoma. Patients with Type G tumors tended to have earlier stage diseases than the other tumor groups.

**Table 2:** Comparison of clinicopathological characteristics

**Variable**	**Type E (SQ) (n = 12)**	**Type E (AD) (n = 6)**	**Type Ge (n = 27)**	**Type G (n = 47)**	***P*-value**
Sex					0.906
Male	10	5	20	37	
Female	2	1	7	10	
Age (mean ± SD)	64.4 ± 6.84	66.3 ± 7.97	65.2 ± 10.6	66.5 ± 9.67	0.728
Extent of surgical resection					< 0.001**
Subtotal esophagectomy with partial gastrectomy	11	3	0	0	
Proximal gastrectomy with partial esophagectomy	1	1	8	20	
Total gastrectomy with partial esophagectomy	0	2	19	27	
Extent of lymph node dissection					< 0.001**
Abdominal, mediastinal and cervical	9	2	0	0	
Abdominal and mediastinal	2	3	4	0	
Abdominal and lower mediastinal^†^	1	1	17	8	
Abdominal	0	0	6	39	
Number of dissected lymph nodes (mean ± SD)	28.1 ± 12.1	28.7 ± 18.1	46.4 ± 34.6	35.3 ± 26.8	0.295
Pathological tumor size (mm, mean ± SD)	46.3 ± 22.4	41.5 ± 36.4	62.2 ± 18.6	37.9 ± 20.5	< 0.001**
Main histological type					< 0.001**
Squamous cell carcinoma	12	0	1	0	
Adenocarcinoma	0	6	26	47	
Esophagogastric junctional invasion					< 0.001**
Yes	6	3	27	0	
No	6	3	0	47	
Siewert classification					< 0.001**
Type I	2	0	0	0	
Type II	1	0	15	0	
Type III	0	0	11	0	
Not applicable	3	12	1	47	
Depth of tumor invasion					0.025*
pT1	3	3	4	23	
pT2	0	1	3	7	
pT3	9	2	14	10	
pT4	0	0	6	7	
Lymph node metastasis					0.005**
pN0	3	3	8	33	
pN1	6	2	6	5	
pN2	2	1	5	6	
pN3	1	0	8	3	
Distant metastasis					< 0.001**
M0	8	5	12	47	
M1	4	1	15	0	
TNM Stage					< 0.001**
pStage I	2	3	4	27	
pStage II	2	0	6	11	
pStage III	4	2	2	9	
pStage IV	4	1	15	0	

* P < 0.05, ** P < 0.01.

^†^ Including lower thoracic paraesophageal, diaphragmatic and posterior mediastinal lymph node.

Incidence of lymph node metastases were summarized in Table 3. Seven (58.3%) of 12 type E (SQ) tumors, 3 (50.0%) of 6 type E (AD) tumors, 19 (70.4%) of 27 type Ge tumors and 14 (29.8%) of 47 type G tumors had lymph nodes metastases (*P* = 0.003). Although incidence of nodal metastasis in pT1 tumor was significantly lower in the type G tumor group than the other type tumor groups, there was no significant difference in pT2, pT3 and pT4 tumors among 4 tumor groups. With regard to lymph node location, no nodal metastasis in the cervical and mediastinal lymph nodes was seen in the type G tumor group. Although nodal metastases in perigastric lymph nodes were seen in all tumor types, only one nodal metastasis in intra-abdominal lymph nodes, except for perigastric lymph nodes, was recognized in type E tumor group. Nodal metastasis at the splenic hilum was seen in only in the Ge tumor group. As a result, incidence rates for nodal metastasis in cervical, mediastinal, and perigastric lymph nodes differed among 4 patients groups.

**Table 3:** Number of patients with positive nodes

**Variable**	**Type E (SQ) (n = 12)**	**Type E (AD) (n = 6)**	**Type Ge (n = 27)**	**Type G (n = 47)**	***P*-value**
Overall	7/12 (58.3%)	3/6 (50.0%)	19/27 (70.4%)	14/47 (29.8%)	0.003**
Depth of tumor invasion					
pT1	2/3 (66.7%)	0/3	2/4 (50.0%)	0/23	0.001**
pT2	–	1/1 (100%)	2/3 (66.7%)	3/7 (42.9%)	0.497
pT3	5/9 (55.6%)	2/2 (100.0%)	9/14 (64.3%)	6/10 (60.0%)	0.697
pT4	–	–	6/6 (100%)	5/7 (71.4%)	0.269
Main histological type					
Squamous-cell carcinoma	7/12 (66.7%)	–	0/1	–	0.462
Adenocarcinoma	–	3/6 (50.0%)	19/26 (73.1%)	14/47 (29.8%)	0.002**
Location of lymph node†					
Cervical LN	2/9 (22.2%)	0/2	–	–	0.655
Upper–middle mediastinal	0/11	0/5	0/4	–	–
Lower mediastinal^‡^	2/12 (16.7%)	2/6 (33.3%)	2/20 (10.0%)	0/8	0.298
Perigastric LN	6/12 (50.0%)	3/6 (50.0%)	17/27 (63.0%)	13/47 (27.7%)	0.026*
Left paracardial	1	2	8	2	
Right paracardial	3	3	10	5	
Lesser curvature	4	1	13	10	
Greater curvature	0	1	4	1	
Suprapyloric	0	0	0	0	
Infrapyloric	0	0	1	0	
LN along left gastric artery	2/12 (16.7%)	1/6 (16.7%)	5/27 (18.5%)	7/47 (14.9%)	0.983
LN at Celiac trunk	0/6	0/3	1/19 (5.3%)	2/24 (8.3%)	0.837
LN along hepatic artery	0/3	0/1	3/19 (15.8%)	1/27 (3.7%)	0.459
LN along splenic artery	0/2	1/3 (33.3%)	2/22 (9.1%)	1/23 (4.3%)	0.356
LN at splenic hilum	–	–	3/17 (17.6%)	0/9	0.262

* P < 0.05; ** P < 0.01.

^†^ Number of the patients with nodal metastasis/number of the patients underwet lymph node dissection (%).

‡ Lower thoracic paraesophageal, diaphragmatic and posterior mediastinal lymph node.

*LN* Lymph node.

Clinicopathological characteristics and clinical courses of seven patients with cervical or mediastinal lymph node metastasis were summarized in Table 4. The location of mediastinal positive nodes was localized in the lower mediastinal area. Six of 7 patients had disease recurrence and 5 patients were deceased. One patient died of another cause without disease recurrence.

**Table 4:** Clinicopathological findings of patients with cervical and mediastinal lymph node metastasis

**Case**	**Tumor type**	**Cervical LN**	**Mediastinal LN**	**Age**	**Sex**	**Tumor size (mm)**	**Distance**^**†**^	**Macroscopic type**	**Histological type**	**pT**	**pN**	**pM**	**Stage**	**Initial recurrence site**	**Status**
1	E (SQ)	SC	–	64	M	50	65	Type 0	SQ (por)	T3	N3	M0	IIIC	LN, lt. adrenal grand	Deceased
2	E (SQ)	SC	LTP	57	M	87	69	Type 0	SQ (por)	T1	N2	M1	IV	LN	Deceased
3	E (SQ)	–	EH	72	M	25	40	Type 2	SQ (mod)	T3	N1	M0	IIIA	LN	Deceased
4	E (AD)	–	EH	73	F	110	100	Type 0	AD (por)	T2	N1	M0	IIB	Peritoneum	Deceased
5	E (AD)	–	LTP, ID	62	M	45	55	Type 2	AD (mod)	T3	N1	M0	IIIA	LN	Deceased
6	Ge	–	LTP	68	M	80	30	Type 1	AD (mod)	T3	N3	M0	IIIC		Deceased (other cause)
7	Ge	–	EH	41	M	65	25	Type 3	AD (por)	T3	N3	M1	IV	LN	Alive with relapse

^†^ Distance between proximal edge of tumor and EGJ in mm.

*AD* adenocarcinoma, *EH* Esophageal hiatus, *ID* Infradiaphragmatic, *LTP* Lower thoracic paraesophageal, *LN* Lymph node, *mod* moderately differentiated. por: poorly differentiated, *SC*, Supraclavicular, *SQ* Squamous-cell carcinoma.

### Surgical outcomes

**Figure 3:**
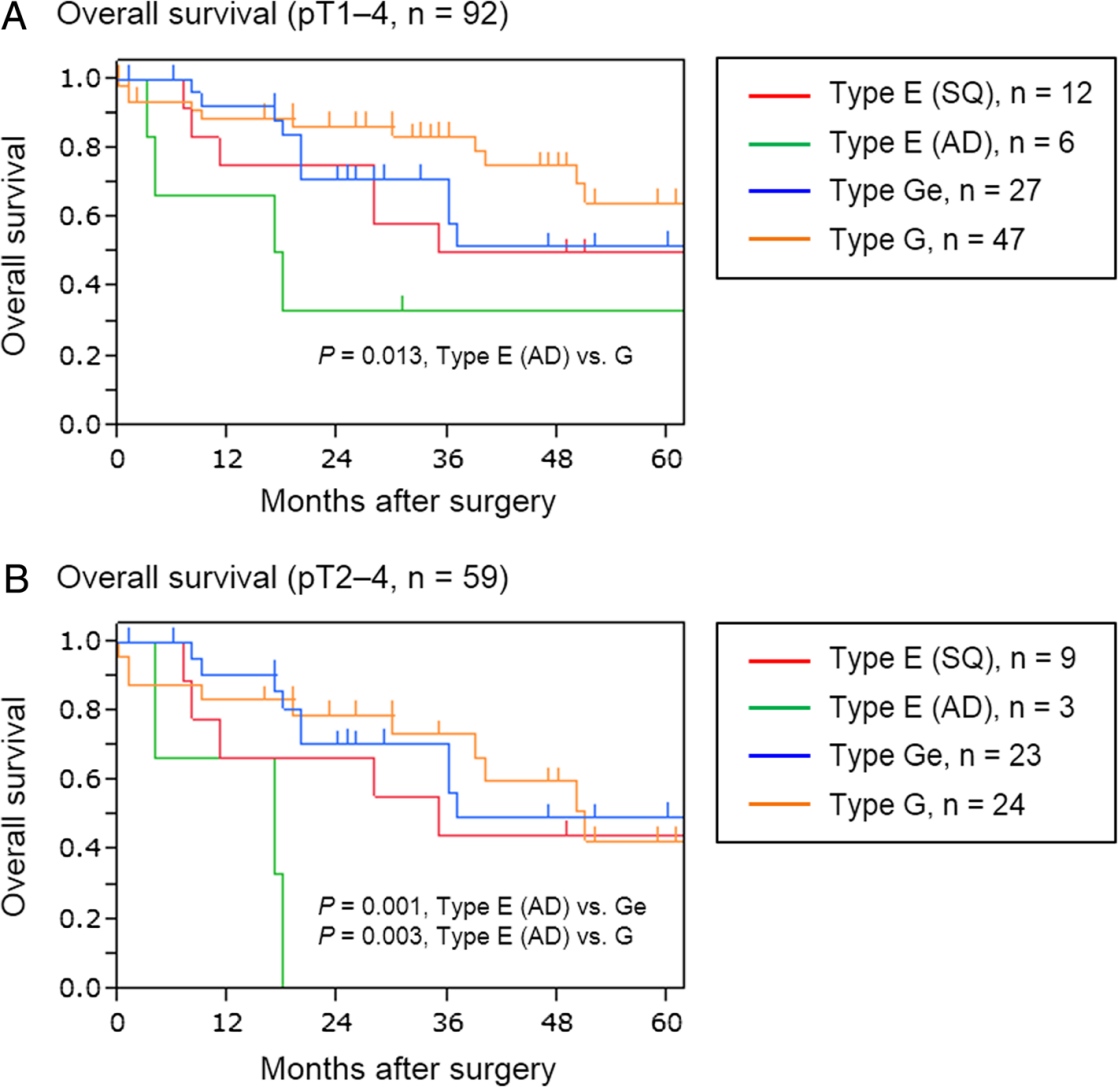
**Overall survival of patients.** (**A**) Patients with pT1–4 tumors (n = 92). Type G tumor group demonstrated higher overall survival rate compared with type E adenocarcinoma (AD) (*P* = 0.013) tumor group. Although not significantly, the type G tumor group had a higher survival rate than the type E squamous-cell carcinoma (SQ) (*P* = 0.366) and Ge (*P* = 0.850) tumor group. (**B**) Patients with pT2–4 Tumors (n = 59). The type E (AD) tumor group demonstrated significantly lower overall survival rate compared with the type Ge (*P* = 0.001) and type G (*P* = 0.003) tumor group. The type E (AD) tumor group had a lower survival rate than the type E (SQ) tumor group (*P* = 0.076) although not significantly.

The 5-year overall survival rate was 56.6%. Thirty-three patients had disease recurrence. Thirty-four patients deceased. Twenty-five, 1 and 8 patients died of cancer, surgical complication and other causes. Overall survival rates were compared among the patients with type E (SQ), E (AD), G and Ge tumors. In patients with pT1–4 tumors, the type G tumor group (overall 5-year survival rate was 64.4%) demonstrated higher overall survival rate compared with type E (AD) (overall 5-year survival rate was 33.3%) (*P* = 0.013) tumor group. Although not significantly, the type G tumor group had a higher survival rate than the type E (SQ) (overall 5-year survival rate was 50.0%) (*P* = 0.366) and Ge (overall 5-year survival rate was 51.9%) (*P* = 0.850) tumor group (Figure 3A). Because the type G tumor group had relatively early-stage disease, survival rates were calculated in patients with pT2–4 tumor. In the pT2–4 group, the type E (AD) tumor group demonstrated significantly lower overall survival rate compared with the type Ge (overall 5-year survival rate was 49.4%) (*P* = 0.001) and type G (overall 5-year survival rate was 42.8%) (*P* = 0.003) tumor group. The type E (AD) tumor group had a lower survival rate than the type E (SQ) tumor group (overall 5-year survival rate was 44.4%) (*P* = 0.076) although not significantly (Figure 3B).

### Prognostic factor

A univariate Cox proportional hazard analysis showed that lymphatic invasion (*P* < 0.001) and venous invasion (*P* < 0.001), depth of tumor invasion (pT category; *P* < 0.001), lymph node metastasis (pN category; *P* < 0.001), distant metastasis (M category; *P* = 0.028) were statistically significant for survival. Sex, age and mail histological type were not significantly associated with survival (Table 5). A multivariate Cox proportional hazard analysis that included variables with *P* < 0.10 in univariate analysis and tumor type (types E (SQ), E (AD), Ge and G) showed that tumor type was an independent significant prognostic factor (Table 6). Among tumor types, the type E (AD) tumor group demonstrated significantly higher risk in survival than did the type E (SQ) (hazard ratio: 0.224; 95% confidence interval, 0.062–0.911; *P* = 0.038), Ge (hazard ratio: 0.162; 95% confidence interval, 0.048–0.643; *P* = 0.012) and G (hazard ratio: 0.219; 95% confidence interval, 0.069–0.839; *P* = 0.029) tumor group. In the depth of tumor invasion, pT1–2 tumor demonstrated significantly lower survival risk than did pT3–4 (hazard ratio: 2.937; 95% confidence interval, 1.168–8.698; *P* = 0.021) tumor. Regarding lymphatic invasion, L1 showed higher survival risk, however there was no significance (hazard ratio: 4.575; 95% confidence interval, 0.940–25.80; *P* = 0.060). Venous invasion, lymph node metastasis (pN category) and distant metastasis (M category) were not significant predictors of survival.

**Table 5:** Univariate Cox proportional hazards analysis of overall survival

**Variable**	**Hazard ratio**	**95%confidence interval**	***P*-value**
Sex			
Male (n = 72)	1.0		
Female (n = 20)	1.391	0.611 – 2.898	0.412
Age (years)			
≤ 65 (n = 38)	1.0		
> 65 (n = 54)	1.141	0.573 – 2.351	0.711
Main histological type			
Squamous-cell carcinoma (n = 13)	1.0		
Adenocarcinoma (n = 79)	0.707	0.323 – 1.769	0.432
Lymphatic invasion			
L0 (n = 32)	1.0		
L1 (n = 60)	7.221	2.558 – 30.22	< 0.001**
Venous invasion			
V0 (n = 32)	1.0		
V1–2 (n = 60)	4.772	1.872 – 16.12	< 0.001**
Depth of tumor invasion			
pT1–2 (n = 44)	1.0		
pT3–4 (n = 48)	4.521	1.993 – 12.14	< 0.001**
Lymph node metastasis			
pN0 (n = 47)	1.0		
pN1–3 (n = 45)	4.597	2.096 – 11.54	< 0.001**
Distant metastasis			
M0 (n = 72)	1.0		
M1 (n = 20)	2.257	1.094 – 4.496	0.028*

* P < 0.05; ** P < 0.01.

*LN* Lymph node.

**Table 6:** Multivariate Cox proportional hazards analysis of overall survival

**Variable**	**Hazard ratio**	**95%confidence interval**	**P-value**
Tumor type			
Type E (AD) (n = 6)	1.0		
Type E (SQ) (n = 12)	0.224	0.062 – 0.911	0.038*
Type Ge (n = 27)	0.162	0.048 – 0.643	0.012*
Type G (n = 47)	0.219	0.069 – 0.839	0.029*
Lymphatic invasion			
L0 (n = 32)	1.0		
L1 (n = 60)	4.575	0.940 – 25.80	0.060
Venous invasion			
V0 (n = 32)	1.0		
V1–2 (n = 60)	0.966	0.196 – 5.170	0.967
Depth of tumor invasion			
pT1–2 (n = 44)	1.0		
pT3–4 (n = 48)	2.937	1.168 – 8.698	0.021*
Lymph node metastasis			
pN0 (n = 47)	1.0		
pN1–3 (n = 45)	1.460	0.463 – 5.607	0.537
Distant metastasis			
M0 (n = 72)	1.0		
M1 (n = 20)	1.097	0.428 – 2.794	0.846

* P < 0.05.

## Discussion

The aim of this study was to clarify the clinicopathological characteristics of cancers around the EGJ, and to investigate optimal management. Standard treatment for EGJC is controversial for several reasons. One of them is that the definition of EGJC is not stable. Siewert et al. define EGJC as adenocarcinoma, centered in area between the lowest 5 cm of the esophagus and the upper 5 cm of the stomach, and crossing the EGJ [14]. The Japanese Classification of Esophageal Cancer (JCEC) from the Japan Esophageal Society defines EGJC as being within the lower 2 cm of the esophagus and the upper 2 cm of the stomach, because of histological evidence of spreading of columnar epithelium-lined lower esophagus [15]. Moreover, AJCC defines EGJ as including squamous-cell carcinoma in the same locations as with Siewert classification [4].

However Siewert classification is widely used, its application is limited for adenocarcinoma. Although EGJC, as defined by the AJCC cancer staging manual, includes squamous-cell carcinoma, it does not categorize any tumor without EGJ invasion as EGJC—as does Siewert classification. Although it estimates prognosis well using different staging systems for squamous-cell carcinoma and adenocarcinoma, this method may be too complex for clinicians; whereas the JCEC system, which treats most limited tumors as EGJC, is more precise.

Because of the unstable definition of EGJCs, clinicopathological characters and treatment strategies have not been unified. Siewert et al. argued that complete surgical resection and lymph node metastasis were independent prognostic factors in type II adenocarcinoma, and subtotal esophagectomy had less survival effectiveness for the patients with type II adenocarcinoma [5]. Hasegawa et al. reported that about 40%, 60% and 90% of patients with type I, II and III tumors, respectively, had lymph node metastases, and recommended complete resection for improving survival [16]. Schiesser et al. reported that subtotal esophagectomy and extended total gastrectomy should be performed for type I and type II–III tumor [17]. With regard to surgical approach, Sasako et al. showed that the left thoracoabdominal approach did not improve survival after the abdominal-transhiatal approach and leads to increased morbidity in patients with cancer of the cardia or subcardia [18]. Kakeji et al. reported that esophagectomy with mediastinal and abdominal lymphadenectomy was adequate for squamous-cell carcinoma, and that extended total gastrectomy with lower mediastinal and abdominal lymphadenectomy was suitable for adenocarcinoma [19]. Carboni et al. maintained effects of extended gastrectomy by an abdominal–trans-hiatal approach for EGJC [20]. Conversely, Chau et al. reported that performance status, liver metastasis, peritoneal metastasis and alkaline phosphatase were independent prognostic factors in patients with locally advanced and metastatic EGJC, and that prognoses of patients with recurrent disease were no better than those without surgery [21].

We studied any tumor centered in area between the lowest 5 cm of the esophagus and the upper 5 cm of the stomach, regardless of histological type and EGJ invasion, and simply categorized them in 4 groups including type E (SQ), E (AD), Ge and G.

Whereas type E (SQ), E (AD) and Ge tumors in this study are categorized as esophageal cancer by AJCC/UICC criteria, these tumor groups show differences in clinicopathological characteristics. In lymph node metastasis, approximately 60%, 50%, 70% and 30% of the patients with type E (SQ), E (AD), Ge and G tumors respectively had lymph node metastases in this study. Cervical lymph node metastases were recognized in only type E (SQ) tumor group. Because type E (AD) tumor was based on columnar epithelium, its histological behavior was thought to be similar to cardiac adenocarcinoma; however, type E (AD) tumor showed a nodal metastatic spreading pattern similar to that of type Ge tumor in this study. Although it seems reasonable to unite type E (AD) and Ge tumors as a group on the basis of lymphadenectomy extent, the patients with type E (AD) tumor showed significantly lower survival rates than other type tumor groups. Although not significantly, patients with type E (AD) tumor had higher incidence of nodal metastasis at mediastinal lymph node than did patients in tumor groups, and all mediastinal positive nodes existed in lower mediastinal area. Thus, subtotal esophagectomy is not necessary for type E (AD) and Ge tumor, if complete tumor resection can be achieved. Because no cervical or mediastinal lymph node metastasis was recognized in the type G tumor group, we should not perform subtotal esophagectomy for type G tumor. In multivariate analysys, tumor type (type E (AD)) was an independent risk factor for survival of the patients with EGJC in this study. The prognosis of cervical or mediastinal node positive patients was poor. Because survival benefit by cervical and mediastinal lymphadenectomy for the node positive patients with EGJC is limited, we should carefully perform subtotal esophagectomy, and cervical and mediastinal lymphadenectomy for EGJC patients. Therefore, extended gastrectomy with or without lower esophagectomy, according to tumor location, and lower mediastinal and abdominal lymphadenectomy is thought to be adequate for patients with EGJC, including type E (SQ) tumor.

Although lymphatic invasion, venous invasion, depth of tumor invasion (T category), lymph node metastasis (N category) and distant metastasis (M category) were significantly prognostic factors in the univariate analysis, tumor type (types E (SQ), E (AD), Ge and G) and depth of tumor invasion (pT3–4 tumor) were significant in the multivariate analysis in this study. It was reported that complete surgical resection and lymph node metastasis were independent prognostic factors in type II adenocarcinoma [5]. We believe that the lack of a significant difference between the prognosis and lymph node metastasis can be explained by limitations of this study such as the small sample size. Distant metastasis (M category) was not significantly prognostic factor in the multivariate analysis in study. AJCC/UICC TNM staging system for esophageal cancer defines nodal metastasis along lesser curvature as distant metastasis, although lymph node along lesser curvature is one of the main regional lymph nodes of gastric cancer. Because majority of the patient with M1 disease had no hematogenous metastasis in this study, there was a possibility that distant metastasis was not significant for prognosis in this study.

Reim et al. reported that chemotherapy to be more efficacious for EGJC than for distal gastric cancer [22]. The treatment efficacy of chemotherapy before or after surgery is unclear in this small scale retrospective cohort study. To clarify optimal treatment strategy for EGJC, we should confirm the results in this study using a large scale prospective study.

## Conclusions

Patients with type E (AD) and Ge tumor had no cervical lymph node metastasis, and those with type G tumor had no nodal metastasis at cervical and mediastinal lymph node. The incidence of mediastinal lymph node metastasis of type E (AD) tumor group was higher than type Ge tumor group, and survival rate of the patients with type Ge tumor is significantly higher than those with type E (AD) tumor. Therefore we should distinguish type Ge tumor from type E (AD) tumor. Based on our findings from a retrospective analysis in this cohort study, we suggest performing extended gastrectomy with or without lower esophagectomy, according to tumor location, and lower mediastinal and abdominal lymphadenectomy for EGJC.

## Competing interests

The authors declare that they have no competing interests.

## Authors’ contributions

HI (Hiroaki Ito)* conceived and designed the study, collected clinical data, and performed the statistical analysis and interpretation of data. HI (Haruhiro Inoue) participated in the study design and performed interpretation of data. NO, HS, MS, SM, YT and HK collected clinical data. SK participated in the study design and coordination. All authors read and approved the final manuscript.

## References

[B1] World Health Organization. International Agency for Research on Cancer GLOBOCAN 2008. Cancer Incidence and Mortality World Wide 2008 [http://globocan.iarc.fr/]

[B2] Pohl H Welch HG The role of overdiagnosis and reclassification in the marked increase of esophageal adenocarcinoma incidence J Nat Cancer Inst 2005 97 142 146 10.1093/jnci/dji024 15657344

[B3] Lu YK Li YM Gu YZ Cancer of esophagus and esophagogastric junction: analysis of results of 1,025 resections after 5 to 20 years Ann Thoracic Surg 1987 43 176 181 10.1016/S0003-4975(10)60391-8 3545110

[B4] Siewert JR Feith M Stein HJ Biologic and clinical variations of adenocarcinoma at the esophago-gastric junction: relevance of a topographic-anatomic subclassification J Surg Oncol 2005 90 139 146 10.1002/jso.20218 15895452

[B5] Siewert JR Stein HJ Feith M Adenocarcinoma of the esophago-gastric junction Scand J Surg 2006 95 260 269 17249275 10.1177/145749690609500409

[B6] Edge SB, Byrd DR, Compton CC AJCC Cancer Staging Manual 2009 7 New York: Springer

[B7] Sobin LH Gospodarowicz MK Wittekind C TNM Classification of Malignant Tumors 2010 7 Oxford: Wiley-Blackwell

[B8] Berger B Stahlberg K Lemminger A Bleif M Belka C Bamberg M Impact of radiotherapy, chemotherapy and surgery in multimodal treatment of locally advanced esophageal cancer Oncol 2011 81 387 394 10.1159/000335263 22269965

[B9] Stahl M Is there any role for surgery in the multidisciplinary treatment of esophageal cancer? Ann Oncol 2010 21 283 285 10.1093/annonc/mdp326 20943629

[B10] Nakajima T Nishi M Kajitani T Improvement in treatment results of gastric cancer with surgery and chemotherapy: experience of 9,700 cases in the Cancer Institute Hospital Tokyo. Sem Surg Oncol 1991 7 365 372 10.1002/ssu.2980070608 1759085

[B11] Peeters KC van de Velde CJ Improving treatment outcome for gastric cancer: the role of surgery and adjuvant therapy J Clinical Oncol 2003 21 272 273 10.1200/JCO.2003.09.136 14645408

[B12] Diazde Liano A Yarnoz C Artieda C Aguilar R Viana S Artajona A Ortiz H Results of R0 surgery with D2 lymphadenectomy for the treatment of localised gastric cancer Clin Translat Oncol 2009 11 178 182 10.1007/S12094-009-0335-9 19293056

[B13] Siewert JR Stein HJ Sendler A Fink U Surgical resection for cancer of the cardia Sem Surg Oncol 1999 17 125 131 10.1002/(SICI)1098-2388(199909)17:2<125::AID-SSU7>3.0.CO;2-9 10449684

[B14] Siewert JR Stein HJ Classification of adenocarcinoma of the oesophagogastric junction British J Surg 1998 85 1457 1459 10.1046/j.1365-2168.1998.00940.x 9823902

[B15] Japan Esophageal Society Japanese Classification of Esophageal Cancer. 10th edition: part I Esophagus 2009 6 1 25 10.1007/s10388-016-0551-7 PMC5222932 28111535

[B16] Hasegawa S Yoshikawa T Cho H Tsuburaya A Kobayashi O Is adenocarcinoma of the esophagogastric junction different between Japan and western countries? The incidence and clinicopathological features at a Japanese high-volume cancer center World J Surg 2009 33 95 103 10.1007/s00268-008-9740-4 18958523

[B17] Schiesser M Schneider PM Surgical strategies for adenocarcinoma of the esophagogastric junction Recent Results Cancer Res 2010 182 93 106 20676874 10.1007/978-3-540-70579-6_8

[B18] Sasako M Sano T Yamamoto S Sairenji M Arai K Kinoshita T Nashimoto A Hiratsuka M Left thoracoabdominal approach versus abdominal-transhiatal approach for gastric cancer of the cardia or subcardia: a randomised controlled trial Lancet Oncol 2006 7 8 644 651 10.1016/S1470-2045(06)70766-5 16887481

[B19] Kakeji Y Yamamoto M Ito S Sugiyama M Egashira A Saeki H Morita M Sakaguchi Y Toh Y Maehara Y Lymph node metastasis from cancer of the esophagogastric junction, and determination of the appropriate nodal dissection Surg Today 2012 42 351 358 10.1007/s00595-011-0114-4 22245924

[B20] Carboni F Lorusso R Santoro R Lepiane P Mancini P Sperduti I Santoro E Adenocarcinoma of the esophagogastric junction: the role of abdominal-transhiatal resection Ann Surg Oncol 2009 16 304 310 10.1245/s10434-008-0247-x 19050964

[B21] Chau I Norman AR Cunningham D Waters JS Oates J Ross PJ Multivariate prognostic factor analysis in locally advanced and metastatic esophago-gastric cancer–pooled analysis from three multicenter, randomized, controlled trials using individual patient data J Clin Oncol 2004 22 2395 2403 10.1200/JCO.2004.08.154 15197201

[B22] Reim D Gertler R Novotny A Becker K Ebert M Dobritz M Langer R Hoefler H Friess H Adenocarcinomas of the esophagogastric junction are more likely to respond to preoperative chemotherapy than distal gastric cancer Ann Surg Oncol 2012 19 2108 2118 10.1245/s10434-011-2147-8 22130620

